# Novel splice isoforms of pig myoneurin and their diverse mRNA expression patterns

**DOI:** 10.5713/ajas.17.0911

**Published:** 2018-04-25

**Authors:** Xiaohong Guo, Meng Li, Pengfei Gao, Guoqing Cao, Zhimin Cheng, Wanfeng Zhang, Jianfeng Liu, Xiaojun Liu, Bugao Li

**Affiliations:** 1 College of Animal Science and Veterinary Medicine, Shanxi Agricultural University, Taigu, Shanxi 030801, China; 2 Key Laboratory of Animal Genetics Breeding and Reproduction, Ministry of Agriculture, College of Animal Science and Technology, China Agricultural University, Beijing 100193, China; 3 College of Animal Science and Veterinary Medicine, Henan Agricultural University, Zhengzhou, 450002, China

**Keywords:** Pig, *MYNN*, Alternative Splicing, mRNA Expression

## Abstract

**Objective:**

The aim of this study was to clone alternative splicing isoforms of pig myoneurin (*MYNN*), predict the structure and function of coding protein, and study temporal and spatial expression characteristics of each transcript.

**Methods:**

Alternative splice isoforms of *MYNN* were identified using RNA sequencing (RNA-seq) and cloning techniques. Quantitative real-time polymerase chain reaction (qPCR) was employed to detect expression patterns in 11 tissues of Large White (LW) and Mashen (MS) pigs, and to study developmental expression patterns in cerebellum (CE), stomach (ST), and *longissimus dorsi* (LD).

**Results:**

The results showed that *MYNN* had two alternatively spliced isoforms, *MYNN-1* (GenBank accession number: KY470829) and *MYNN-2* (GenBank accession number: KY670835). *MYNN-1* coding sequence (CDS) is composed of 1,830 bp encoding 609 AA, whereas *MYNN-2* CDS is composed of 1,746 bp encoding 581 AA. *MYNN-2* was 84 bp less than *MYNN-1* and lacked the sixth exon. MYNN-2 was found to have one C_2_H_2_ type zinc finger protein domain less than MYNN-1. Two variants were ubiquitously expressed in all pig tissues, and there were significant differences in expression of different tissues (p<0.05; p<0.01). The expression of *MYNN-1* was significantly higher than that of *MYNN-2* in almost tissues (p<0.05; p<0.01), which testified that *MYNN-1* is the main variant. The expression of two isoforms decreased gradually with increase of age in ST and CE of MS pig, whereas increased gradually in LW pig. In LD, the expression of two isoforms increased first and then decreased with increase of age in MS pig, and decreased gradually in LW pig.

**Conclusion:**

Two transcripts of pig *MYNN* were successfully cloned and *MYNN-1* was main variant. *MYNN* was highly expressed in ST, CE, and LD, and their expression was regular. We speculated that *MYNN* plays important roles in digestion/absorption and skeletal muscle growth, whereas the specific mechanisms require further elucidation.

## INTRODUCTION

The BTB (Broad complex, Tramtrack, Bricabrac) domain superfamily proteins have many functions such as activation and repression of transcription, cytoskeleton organization, and chromatin remodeling [1]. The BTB protein is not a traditional transcriptional regulator, as it functions as both a transcriptional activator and repressor [2–5]. It functions through rotation and reconstruction on the nucleosome to activate transcription [6]. It can also inhibit transcription factors (e.g., histone lysine-specific demethylase, promyelocytic leukemia zinc finger and B-Cell CLL/Lymphoma 6) [7–9] leading to inhibition of the transcriptional activity of related genes, mainly by the recruitment of transcriptional corepressors (e.g., nuclear receptor corepressor and its homolog SMRT, mSi3a [component of histone deacetylase complex], histone deacetylase) via its homodimeric domain [10–12]. The zinc finger protein is a kind of common transcriptional regulatory protein, which can regulate the expression of target genes mainly by combining with specific gene sequence, and combining with its own or other zinc finger protein. So then adapt to the development, differentiation and maturation to of organism at different stages of individual [13]. The study found that about 5% to 10% of C_2_H_2_ zinc finger proteins contain the BTB domain at the N end [14], which forms a new class of transcription factor BTB/POZ and zinc finger proteins (BTB/POZ-ZF) family, which usually act with other transcription factors or cofactors to form a transcriptional complex that activates or inhibits transcription. Myoneurin (MYNN) belongs to the BTB/POZ-ZF family, which contains an amino-terminal BTB/POZ domain and eight tandem repeated zinc-finger motifs of the C_2_H_2_-type (zinc finger, C_2_H_2_-type) [14,15], and plays an important role in the regulation of gene expression.

MYNN, identified in the mouse embryo, is highly homologous to the human protein [16]. Promoter beta-galactosidase constructs were used to define the region (first intron) that conferred basal expression of the *MYNN* gene [16]. The expression of *MYNN* was found to be abnormal during nerve crush or axotomy in extrasynaptic myonuclei, which proved that MYNN is a likely candidate to mediate electrical activity-dependent expression of downstream synapse-specific genes [14]. MYNN was identified within the peripheral condensed chromatin and euchromatin/heterochromatin regions by electron microscopy analyses, and as such, may play an important role in chromatin structure and transcription [14]. MYNN can inhibit transcription in neuromuscular tissue, and its expression increased after denervation; therefore, it may regulate the expression of synaptic genes [14]. Studies have shown that MYNN represses gene transcription by recruiting a transcriptional corepressor and histone deacetylase [16]. MYNN expression in ovarian serous adenocarcinoma and lung (LU) squamous cell carcinoma was found to be as high as 34.1% and 33.3% respectively. In addition, MYNN is a potential promoting factor in head and neck squamous cell carcinoma, endometrial carcinoma, gastric carcinoma, and bladder epithelial cell carcinoma [17].

*MYNN* is widely expressed in various tissue types in mammals [18–20]. Northern blot analysis showed that in humans the expression of a major 2.5 kb MYNN in muscle was higher than that in other tissues including the testis, ovary, and placenta [15] The expression of MYNN was detected in the cerebellum (CE), skeletal muscle, testis, heart (H), brain, and liver (LI) tissues in the mouse [15]. Muscle tissue is a primary site of *MYNN* gene transcription, and the expression of nuclear *MYNN* is associated with subsynaptic nuclei at the neuromuscular junction, and is also developmentally regulated during the late embryonic period (E16, P0) [14]. By analyzing the overall structure of MYNN and examining its expression in human muscle, Alliel et al [15] showed that MYNN can regulate gene expression in the muscle. To date, there are no study on the expression patterns of *MYNN* in pig, especially its alternative splice variants.

The Mashen (MS) pig is a local breed in North China. It is hardy, adaptable to mountainous environments, and has good meat quality. However, the growth rate and lean/fat ratio are much lower than those in Western commercial pig breeds such as Large White (LW) and Landrace [21–23]. Previous studies have demonstrated the function of MYNN in the human and mouse, but no data have been reported about pig MYNN. The aim of this study was to identify the alternatively spliced transcript variants of pig *MYNN* using RNA sequencing (RNA-seq) results, and to measure their mRNA expression profiles to provide a foundation for further studies on this protein.

## MATERIALS AND METHODS

All of the animal procedures were as per the Code of Ethics of the World Medical Association (Declaration of Helsinki) for animal experiments (http://ec.europa.eu/environment/chemicals/lab_animals/legislation_en.htm). The methods were performed in accordance with the Good Experimental Practices adopted by the College of Animal Science and Veterinary Medicine, Shanxi Agricultural University (Shanxi, China). Moreover, the local animal welfare laws, guidelines, and policies were strictly followed for the feed and use of experimental animals.

### Animal and sample collection

In this study, a total of nine healthy LW and nine healthy MS male pigs were selected from the Datong Pig Breeding Farm (Shanxi, China). All of the animals were kept under the same feeding and environmental conditions. They were weaned at 28 days old and were castrated when weaning. Three pigs of each breed were slaughtered at each of the three development stages at 1, 90, and 180 days after birth. Eleven different tissues including H, LI, spleen (SP), LU, kidney (K), CE, small intestine (SI), stomach (ST), pancrease (P), *longissimus dorsi* (LD), and fat (F) were collected, immediately snapped in liquid nitrogen, and stored at −80°C for subsequent use. The transcriptome of LD at each of the three development stages was subjected to RNA-seq analysis.

### RNA extraction and purification

Total RNA was extracted using TRIZOL reagent (Life Technologies, Carlsbad, CA, USA) following the manufacturer’s instructions. RNA integrity number (RIN) was checked to determine RNA integrity using an Agilent Bioanalyzer 2100 (Agilent Technologies, Santa Clara, CA, USA). All of the solutions had a RIN ≥7.0 and 28S/18S ≥0.7. Total RNA was further purified using the RNeasy Micro Kit (QIAGEN GmBH, Hilden, Germany) and RNase-Free DNase Set (QIAGEN, Germany).

### Sequencing and comparative analysis

RNA (2 μg) was depleted of ribosomal RNA (Beckman Coulter, Beverly, MA, USA). First strand cDNA was synthesized using SuperScript II Reverse Transcriptase, and second strand cDNA synthesis was subsequently performed using DNA polymerase I and RNase H. The library fragments were purified with AMPure XP system (Beckman Coulter, USA). Thereafter, 2.5 μL USER Enzyme (NEB, Ipswich, MA, USA) was added to size-selected, adaptor-ligated cDNA, and the mixture was incubated at 37°C for 15 min followed by 5 min at 95°C. Subsequently, polymerase chain reaction (PCR) was performed with Phusion High-Fidelity DNA polymerase, Universal PCR primers, and Index (X) primer. The PCR products were purified (AMPure XP system; Beckman Coulter, USA) and the library quality was assessed on the Agilent Bioanalyzer 2100 system. The clustering of the index-coded samples was performed on a cBot Cluster Generation System using TruSeq PE Cluster Kit v4-cBot-HS (Illumina, San Diego, CA, USA) according to the manufacturer’s instructions. After cluster generation, the library preparations were sequenced on an Illumina Hiseq 2500 platform to generate 2×100 base pair (bp) paired-end reads.

Raw data (raw reads) in fasta format were first processed through in-house perl scripts. In this step, clean data (clean reads) were obtained by removing the reads containing the adapter sequence, reads containing poly-N, and low-quality reads from raw data. At the same time, Q20, Q30, GC content, and sequence duplication level of the clean data were calculated. All of the downstream analyses were based on clean data of high quality. Then these clean reads were mapped to the pig genome (UCSC susScr3, from the UCSC Genome Browser [ftp://hgdownload.cse.ucsc.edu/goldenPath/susScr3/bigZips/susScr3.fa.gz]). Only the reads with a perfect match or one mismatch were further analyzed and annotated based on the reference genome. TopHat2 software was used to map the reference genome [24,25].

### Prediction of alternative splicing

Alternative splicing with less than 5% reads were filtered out. Then the alternative splicing sites were mapped to known splicing sites (1 bp error was allowed) to identify known alternative splicing sites. Finally, novel unmapped alternative splicing sites were classified.

### Identification of pig *MYNN* alternative splice variants

**Figure 1:**
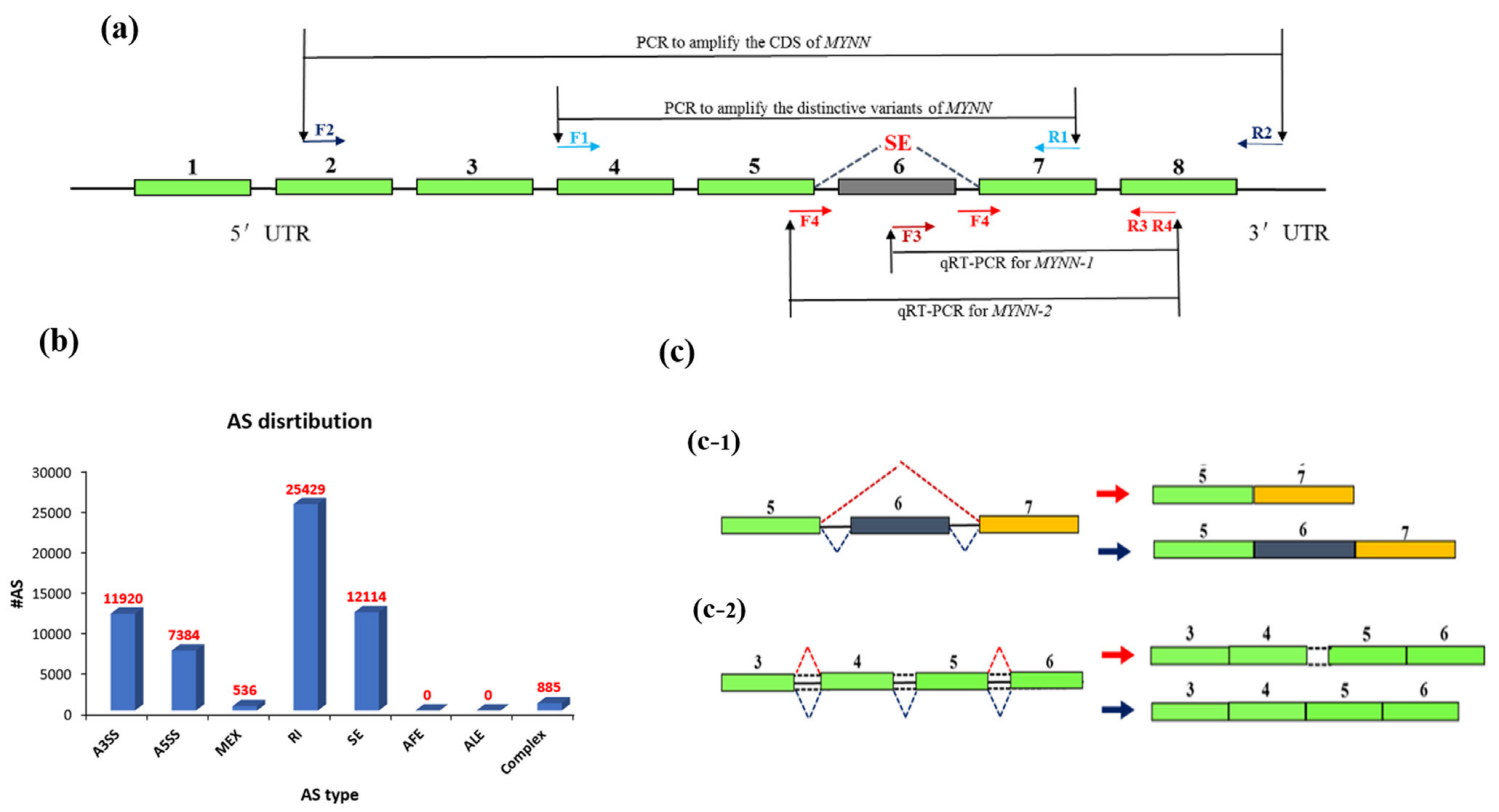
Genomic structure diagram of pig myoneurin (*MYNN*). (a) Cloning strategy for pig *MYNN* cDNA; (b) Classification of *MYNN* alternative splicing; (c) Genomic structure diagram of two alternative splice variants is predicted.

Based on the results of alternative splicing in RNA-seq, the primers in this experiment were designed using the NCBI Nucleotide System and then tested using Oligo6 software. Two primers were designed to amplify the distinctive variants and two primers were used for quantitative real-time PCR (qPCR), as shown in Table 1. The strategy is shown in Figure 1a. PCR reactions were performed in a 25 μL reaction volume containing 50 ng cDNA Pool (18 muscle tissues were used for sequencing), 0.5 μM of each primer, 12.5 μL of 2×Taq PCR MasterMix (TIANGEN, Beijing, China), and ddH_2_O to a total volume 25 μL. The PCR reaction conditions were as follows: denaturation at 94°C for 5 min followed by 35 cycles at 94°C for 30 s, 60°C for 30 s, and 72°C for 30 s, with a final extension at 72°C for 5 min. The PCR products were examined by electrophoresis on a 1.0% agarose gel.

### Cloning of *MYNN* alternative splice variants and bioinformatics analysis

PCR products were purified using a Gel Extraction Kit (Sangon Biotech, Shanghai, China) and ligated into the pMD19-T Vector (Takara, Dalian, China) for sequencing. Nucleic acid sequences were aligned using the NCBI Nucleotide Blast database (http://www.ncbi.nlm.nih.gov/blast) and DNAMAN 7 software. MegAlign software was used to align the amino acids of MYNN among 10 species to calculate their homology. Phylogenetic trees were constructed based on the neighbor-joining method in MEGA4 and NCBI pairwise alignments (https://blast.ncbi.nlm.nih.gov/Blast.cgi).

### Measurement of *MYNN-1* and *MYNN-2* mRNA expression levels

To determine whether differentially expressed *MYNN* is involved in the development of muscle, we used qPCR to analyze expression of the two *MYNN* alternative splice variants in the LD of LW and MS pigs at three time points (1 d, 90 d, and 180 d). In addition to muscle, total RNA was extracted from 10 additional tissues of LW and MS pigs, including H, LI, SP, LU, K, CE, SI, ST, P, and F, at 90 d. Specific primers for validation and spatial expression analysis of the MYNN alternative splice variants are shown in Table 1. Total RNA was extracted using Invitrogen Ambion TRIzol LS Reagent (Life Technologies, USA). The cDNA was synthesized by reverse transcription from 500 ng total RNA using the PrimeScript RT Reagent Kit with gDNA Eraser (Takara, China) according to the manufacturer’s instructions. The qPCR was performed using a SYBR PrimeScript RT-PCR Kit (Takara, China) performed on an ABI-7500 (Life Technologies, USA) under the following conditions: pre-denaturation at 95°C for 30 s, 45 cycles of 95°C for 5 s and 60°C for 34 s, one cycle of 95°C for 15 s, 60°C for 1 min, and 95°C for 30 s. All of the qPCR reactions for each gene were performed using three biological replicates, with three replicates per experiment.

### Statistical analysis

Average threshold (Ct) values per triplicate were used to calculate the relative amounts of mRNA using the 2^−ΔΔCt^ method, and *18S rRNA* was used as an internal gene. Statistical differences between the expression levels of *MYNN* splice variants were determined with analysis of variance (ANOVA) using SPSS 22.0. Differences between LW and MS pigs were analyzed using the Student’s *t*-test, and differences among different time points were identified using ANOVA.

## RESULTS

### RNA-seq results

Illumina sequencing yielded approximately 2,718 million paired clean reads (100 nucleotides [nt]). The sequenced fragments were mapped to the pig genome in UCSC (susScr3) using TopHat software, which can align reads across splice junctions without relying on gene annotation. More than 73% of the total reads (2,793 million) were mapped to the pig genome. The results of TopHat included all alternative splicing information, which was classified as skipped exon (SE), alternative 5′ splicing site, alternative 3′ splicing site, retained intron (RI), mutually exclusive exon, alternative promoter, alternative polyadenylation. The results are shown in Figure 1b.

### Prediction of sites and types of alternative splice variants of *MYNN* in pig muscle

**Figure 2:**
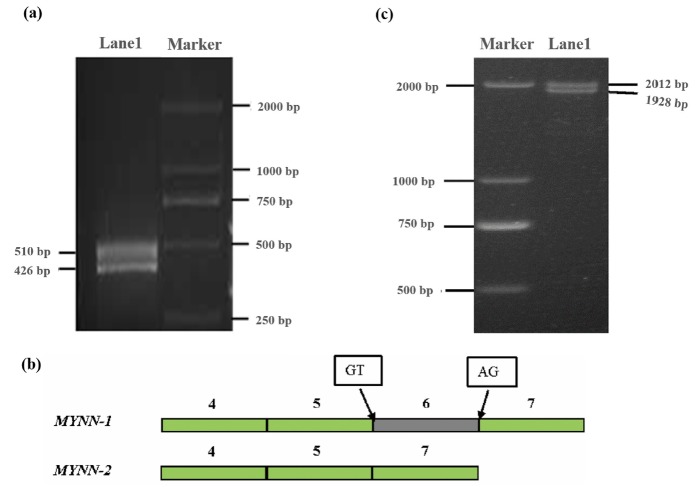
Products amplified by the P1 and P2 primers. (a) Products amplified by the P1 primer; (b) Coding sequence structure diagram of *MYNN*-1 and *MYNN*-2; (c) Products amplified by the P2 primer. *MYNN*, myoneurin.

Two types of MYNN alternative splice variants were predicted in pig muscle. An SE was predicted at the sixth exon (Figure 1c-1), and a RI was present at the fourth and fifth exons, which corresponded to the fourth intron (Figure 1c-2).

### Identification of alternative splice variants of pig *MYNN* in muscle tissue

Two fragments of 510 bp and 426 bp were amplified by primer P1 (Figure 2a), and were named *MYNN-1* and *MYNN-2*, respectively. Interestingly, after sequencing and alignment, we found that *MYNN-2* lacked the sixth exon compared with *MYNN-1* (Figure 2b). The splice sites complied with the GT-AG rules at the 5′ splice donor and 3′ splice acceptor sites (Figure 2b).

Using a pool of cDNA as the template, the coding sequence (CDS) of *MYNN-1* and *MYNN-2* was amplified using P2 primers, leading to the successful amplification of two fragments of 2,012 bp and 1,928 bp (Figure 2c). The results of sequencing and alignment were in agreement with the alternative splice variants predicted by RNA-seq. *MYNN-1* (GenBank accession number: KY470829) is 84 bp longer than *MYNN-2* (GenBank accession number: KY670835), which further proved that there were two alternative splice variants; no other variants were found in the pig muscle tissues. The CDS of the pig *MYNN-1* and *MYNN-2* variants is 1,830 bp and 1,746 bp respectively, *MYNN-2* lacked the sixth exon than *MYNN-1*, as shown in Supplementary Figure S1. Pig *MYNN-1* CDS is composed of 1,830 bp encoding 609 amino acids, whereas *MYNN-2* CDS is composed of 1,746 bp encoding 581 amino acids.

### Bioinformatics analysis

The prediction of function domain found MYNN-1 contains an amino-terminal BTB/POZ domain at the N end and eight tandem repeated zinc-finger motifs of the C_2_H_2_-type at the C end. Nevertheless, MYNN-2 also contains a BTB domain at the N end, and differs from MYNN-1 in that it less a zinc finger protein domain at the C end (Supplementary Figure S2).

**Figure 3:**
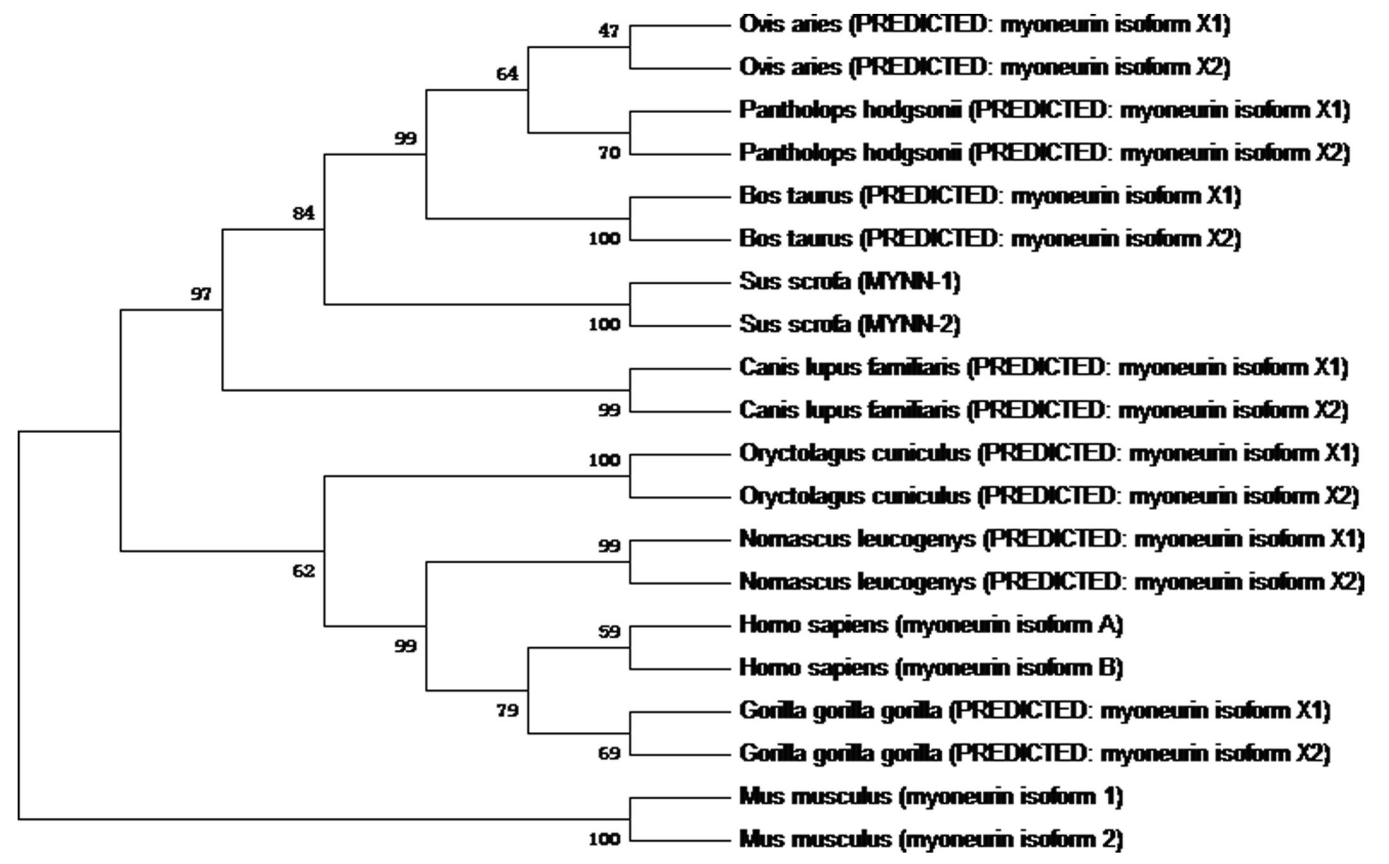
Phylogenetic tree of myoneurin (MYNN) amino acid sequences in 10 species. Ovis aries (XP_004003228.1; XP_011955094.1); *Pantholops hodgsonii* (XP_005964586.1; XP_005964587.1); *Bos taurus* (XP_005201749.1; XP_005201752.1); *Canis lupus familiaris* (XP_861907.2; XP_005639965.1); *Oryctolagus cuniculus* (XP_008264687.1; XP_002716433.1); *Nomascus leucogenys* (XP_012366261.1; XP_012366262.1); *Homo sapiens* (NP_001172047.1; NP_001172048.1); *Gorilla gorilla gorilla* (XP_004038031.1; XP_018880272.1); *Mus musculus* (NP_085034.2; NP_001276550.1).

MegAlign software was used to determine the homology of MYNN amino acid sequences of 10 species including the pig, human, and sheep. Phylogenetic trees (as shown in Figure 3) of 10 species were constructed by the neighbor-joining method using Mage 7.0. The results showed that the MYNN-1 amino acid sequence was 97% homologous with *Bos Taurus* isoform X1, *Canis lupus familiaris* isoform X1, *Pantholops hodgsonii* isoform X1 and *Ovis aries* isoform X1. The homology of *Sus scrofa* MYNN-1 amino acid sequence with *Pantholops hodgsonii* isoform X2, *Sus scrofa* MYNN-2, *Oryctolagus cuniculus* isoform X1, *Homo sapiens* isoform X1, *Gorilla gorilla gorilla* isoform X1 and *Nomascus leucogenys* isoform X1 was 96%, 95%, 95%, 95%, 95%, and 95%, respectively. The MYNN-2 amino acid sequence was most homologous with *Canis lupus familiaris* isoform X2 and *Ovis aries* isoform X2, with homology of 97%. *Mus musculus* MYNN, as another branch, was distant with the other nine species. The MYNN development tree is consistent with the evolution of species, which suggests that MYNN exhibits similar functions in different species.

### Tissue expression patterns of *MYNN-1* and *MYNN-2*

**Figure 4:**
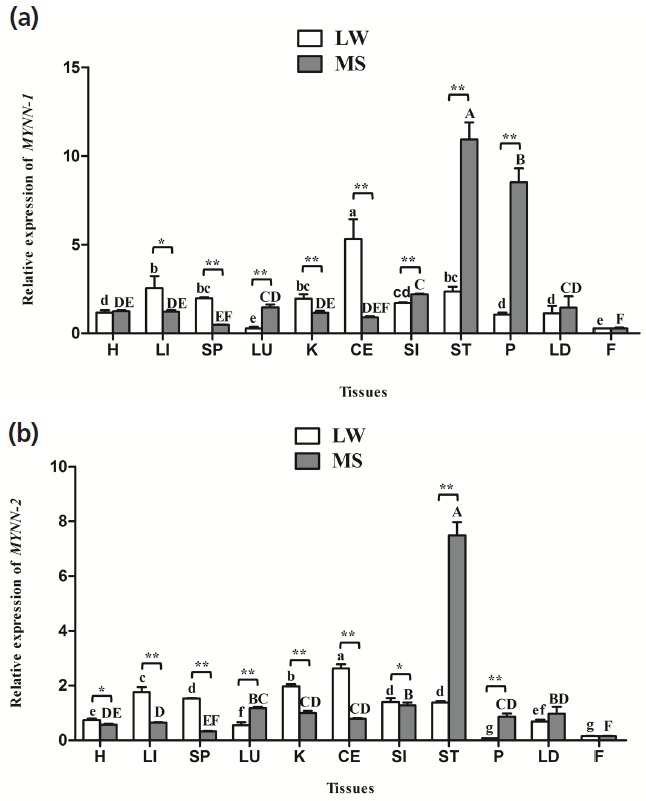
*MYNN* mRNA expression in pig tissues. (a) Relative mRNA expression of *MYNN-1* in different tissues of LW and MS pigs; (b) Relative mRNA expression of *MYNN-2* in different tissues of LW and MS pigs. *MYNN*, myoneurin; LW, Large White pig; MS, Mahsen pig; H, heart; LI, liver; SP, spleen; LU, lung; K, kidney; CE, cerebellum; SI, small intestine; ST, stomach; P, pancreas; LD, *longissimus dorsi*; F, fat. Values in the bar diagram indicate “mean±standard errors”. In the same breed, means with no common letter differ significantly. In the same tissue, “*” indicates that the difference between LW and MS had a significance level of 0.05, “**” indicates that the difference between LW and MS had a significance level of 0.01.

*MYNN-1* and *MYNN-2* were widely distributed in all of the tissues, and there were significant differences in expression in different tissues (Figure 4). Notably, the two variants tended to have higher expression levels of ST, LU, and P in MS pig than in LW pig (p<0.01). Nevertheless, their expression levels in the CE, K, LI, and SP of LW pig were significantly higher than in the MS pig (p<0.01, p<0.05); The relative expression of *MYNN-1* and *MYNN-2* was higher in CE, LI, K, and was lowest in F in LW pig. Meanwhile, their expression levels was higher in ST, P, SI, and was lowest in F in MS pig; The expression level of *MYNN-1* was significantly higher than *MYNN-2* in almost tissues in both breeds (p<0.01, p<0.05).

### Developmental expression profile of *MYNN-1* and *MYNN-2* in CE

**Figure 5:**
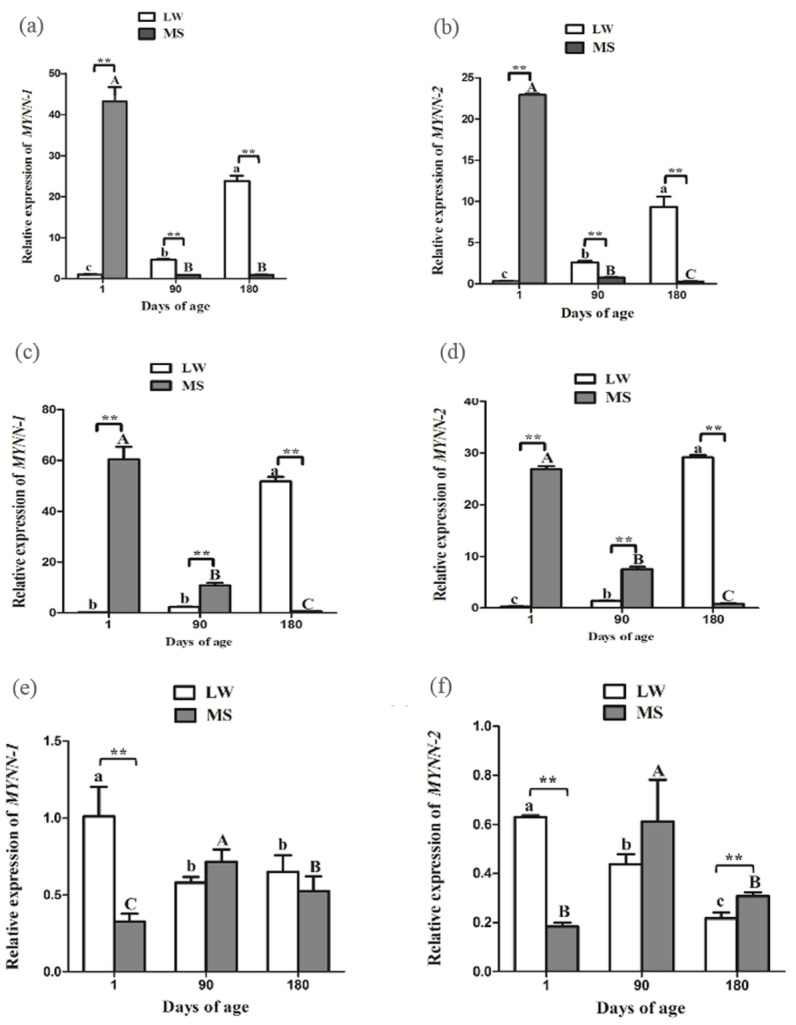
*MYNN-1* and *MYNN-2* expression in CE, ST, and LD of LW and MS pigs at different time points. (a) Relative mRNA expression of *MYNN-1* in LW and MS pigs at different stages; (b) Relative mRNA expression of *MYNN-2* in LW and MS pigs at different stages; (c) Relative mRNA expression of *MYNN-1* in LW and MS pigs at different stages; (d) Relative mRNA expression of *MYNN-2* in LW and MS pigs at different stages; (e) Relative mRNA expression of *MYNN-1* in LW and MS pigs at different stages; (f) Relative mRNA expression of *MYNN-2* in LW and MS pigs at different stages. *MYNN*, myoneurin; CE, cerebellum; ST, stomach; LD, *longissimus dorsi*; LW, Large White pig; MS, Mahsen pig. In the same breed, means with no common letter differ significantly. In the same tissue, “**” indicates that the difference between LW and MS had a significance level of 0.01.

During the growth and development of LW and MS pigs, *MYNN-1* and *MYNN-2* exhibited similar expression patterns in CE (Figure 5a, 5b). The two variants tended to have higher expression levels in MS pig than in LW pig at 1 d time point (p<0.01). Nevertheless, their expression levels of LW pig were significantly higher than in MS pig at other time points (p< 0.01); in the LW pig, the expressions of *MYNN-1* and *MYNN-2* increased gradually with the increase of age, whereas, the expressions of *MYNN-1* and *MYNN-2* decreased gradually in MS pig; *MYNN-1* expression was significantly higher than that of *MYNN-2* at different stages in each breed (p<0.01, p< 0.05).

### Developmental expression profile of *MYNN-1* and *MYNN-2* in ST

In ST, the two variants tended to have higher expression levels in LW pig than in MS pig at 180 d time point (p<0.01) (Figure 5c, 5d). Nevertheless, their expression levels of LW pig were significantly higher than in MS pig at other time points (p< 0.01); In LW pig, the expressions of *MYNN-1* and *MYNN-2* increased gradually with the increase of age, whereas, the expressions of *MYNN-1* and *MYNN-2* decreased gradually in MS pig; *MYNN-1* expression was significantly higher than that of *MYNN-2* at almost stages in each breed (p<0.01).

### Developmental expression profile of *MYNN-1* and *MYNN-2* in LD

In LD, the two variants tended to have higher expression levels in MS pig than in LW pig at 1 d time point (p<0.01) (Figure 5e, 5f); In the LW pig, the two variants were significantly more highly expressed in the LD at 1 d, whereas, the expression was highest at 90 d in the MS pig; With the exception of the 90 d time point in MS pig, *MYNN-1* expression was significantly higher than that of *MYNN-2* at different stages in each breed (p<0.01, p<0.05).

## DISCUSSION

Alternative splicing refers to an pre-mRNA through different splicing, ultimately resulting in different structural and functional protein isoforms [26]. Alternative splicing is widespread in the body, and is related to changes in protein function [27]. Using a new generation of high-throughput sequencing technology, RNA-seq being used to discover and identify alternatively spliced events in animals and plants for which can accurately quantify gene expression in tissues and identify gene sequences accurately [28,29]. Mortazavi et al [30] detected 3,500 splicing variants in mice by RNA-seq, and found that there were 145,000 mRNA splicing forms. Graveley et al [29] detected multiple developmental stages of transcription in Drosophila melanogaster by RNA-seq technology. The results showed that 12,295 exons were found have 22,965 new splice sites, and more than half of them were variable splicing [31]. This experiment was based on the sequencing results of RNA-seq to study the alternative splicing of pig *MYNN* gene, and we obtained two novel alternative isoforms of pig *MYNN* for the first time. *MYNN-1* CDS is composed of 1,830 bp encoding 609 amino acids, *MYNN-2* CDS is composed of 1,746 bp encoding 581 amino acids.

The expression levels of each transcript of the same gene is different, and the transcript with highest expression is known as the main isoform, whereas those with relatively few transcripts are called secondary isoforms [32]. In our study, the mRNA expression level of *MYNN-1* was significantly higher than *MYNN-2* in most tissues (p<0.01, p<0.05), meanwhile, the expression of *MYNN-1* in the CE, ST, LD were significantly higher than *MYNN-2* at most stages. Thus, it is clear that *MYNN-1* is the main variant in pig (p<0.01, p<0.05).

Differences in gene expression are closely related to physiological functions, so evaluation of gene expression characteristics provided the basis for studying the function of *MYNN-1* and *MYNN-2* genes. Previous studies have found that BTB/POZ protein is widely expressed in various tissues of mammals [20–22]. In humans, the expression of a 2.5 kb MYNN was found to be more highly expressed in muscle than in other tissues, and was detected in the cerebellum, skeletal muscle, testis, hearth, brain, and LI tissues of the mouse [17]. The results of our study showed that *MYNN-1* and *MYNN-2* were ubiquitously but differentially expressed in the H, LI, SP, LU, K, CE, SI, ST, P, LD, and F, which were basically consistent with previous studies (p<0.05). The universal expression of the *MYNN* gene is consistent with its function as a transcription factor to regulate gene transcription. Further analysis showed that *MYNN-1* and *MYNN-2* were highest expressed in CE in LW pig, which proved that *MYNN* may relate to the development of CE. Those two isoforms were highly expressed in the ST, P, and SI of MS pig, and the ST, P, and SI all are main digestive organs. Therefore, we hypothesized that *MYNN* is closely related to the digestion and absorption of MS pig. In addition, these two isoforms of different expression characteristics in different breeds may be caused by the different genetic background of LW pig and MS pig.

Therefore, we further studied the developmental expression profile of *MYNN-1* and *MYNN-2* in ST, the result showed that: The expression of *MYNN-1* and *MYNN-2* decreased gradually with the increase of age in MS pig. The gastrointestinal tract are not fully developed when piglets was born. Its weight and volume were relatively small, the secretion of digestive enzymes was insufficient, the digestion and absorption function was not complete, such as pepsin exists in the state of zymogen in piglet birth, which can not digest protein, especially vegetable protein. The piglets at 1 day have a ST weight of only 4 to 8 g, which can hold only about 40 mL of milk. The gastrointestinal tract of piglets developed rapidly during the whole lactation period, and pepsin began to have digestive function at 35 to 40 days. The digestive function gradually completed at 75 days old, and the gastrointestinal tract was no longer developed at 180 days. It indicted that the developmental expression profile of *MYNN-1* and *MYNN-2* in ST were consistent with gastrointestinal tract in MS pig. Nevertheless, for the LW pig, these two isoforms showed a downward trend with the increase of the age, so we infered LW pig *MYNN* had the opposite effect with MS pig.

By analyzing the overall structure of MYNN and examining its expression in human muscle, Alliel et al [33] showed that MYNN can regulate gene expression in the muscle. In our study, *MYNN-1* and *MYNN-2* had lowest expression in F and moderate expression in LD compared with other tissues in both LW and MS pigs. Therefore, to further study the expression patterns of MYNN in muscle tissue, we examined the mRNA expression of *MYNN-1* and *MYNN-2* in different developmental stages in both breeds. Interestingly, the gene expression trends of *MYNN-1* and *MYNN-2* were similar in the splice variants. *MYNN-1* and *MYNN-2* were most highly expressed in the LD at 1 d compared to other stages in the LW pig. It is generally believed that the number of muscle fibers will no longer change after the birth of an animal, and that the muscle growth depends mainly on the thickening of the muscle fiber rather than the increase in the number [33]. The development of muscle fiber basically concentrated in the period, which from pregnancy until 2 month old after birth of two months, suggesting *MYNN* has the same development trend as skeletal muscle fiber. The expression of *MYNN-1* and *MYNN-2* increased first and then decreased with the increase of age for MS pig. The development of skeletal muscle is regulated by a series of myogenic determinants. The myogenic determining factor (*MyoD*) and myogenin (*MyoG*) genes were belong to the myogenic regulatory family, which is involved in the formation of muscle fiber and is a candidate gene for muscle growth and meat quality [34]. Our research group previous studied and found that *MyoD* showed the trend of first rising and then decreasing at the 1 to 180 days of age, and the expression of *MyoG* was significantly lower at 1 and 180 days, and reached the highest value at 90 days [34].The developmental expression profile of *MYNN-1* and *MYNN-2* were the basically same with those two myogenic factor.

## CONCLUSION

In this paper, two transcripts of *MYNN* (*MYNN-1* and *MYNN-2*) were identified in muscle tissue of pigs for the first time. And *MYNN-1* was main variant. *MYNN* was highly expressed in ST, CE, and LD, and their expression were regular. We speculated that *MYNN* played important roles in digestion/absorption and skeletal muscle growth, whereas the specific mechanisms were still remaining to be further elucidated.

## Supplementary Data



## Figures and Tables

**Table 1:** PCR primer sequences of the pig *MYNN* gene for amplification

Names	Primer sequences (5′→3′)	Product sizes (bp)	Annealing (Tm, °C)	Notes
P1	F1:GTGAGAAGCCATACAAATGTGAA R1:CAGAATGGACTTTTGTTTTGTGC	426	60	Clone
P2	F2:AGAACAAGGGTAAAATTCGTTTGTG R2: TGCAGCATCAGGTGCTTTTA	1928	60	Clone
P3	F3: CCTCAGGAGAGCTCAACAAACA R3: TGGACTCTTTTTCACTCAAGGGAT	186	60	qPCR for MYNN-1
P4	F4:CACTCATTCTCGAAAACATACAGGA R4: TGGACTCTTTTTCACTCAAGGGAT	170	60	qPCR for MYNN-2
18S	F:CCCACGGAATCGAGAAAGAG R: TTGACGGAAGGGCACCA	132	60	qPCR for 18S rRNA

PCR, polymerase chain reaction; *MYNN*, myoneurin; qPCR, quantitative real-time PCR.
