# Supplementary material for: Novel splice isoforms of pig myoneurin and their diverse mRNA expression patterns
Source: Asian-Australas J Anim Sci. 2018 Apr 25;31(10):1581–90. doi: 10.5713/ajas.17.0911 (PMC6127594; doi:10.5713/ajas.17.0911)
Supplement: Supplementary file 1 [file ajas-31-10-1581-supplementary.pdf]

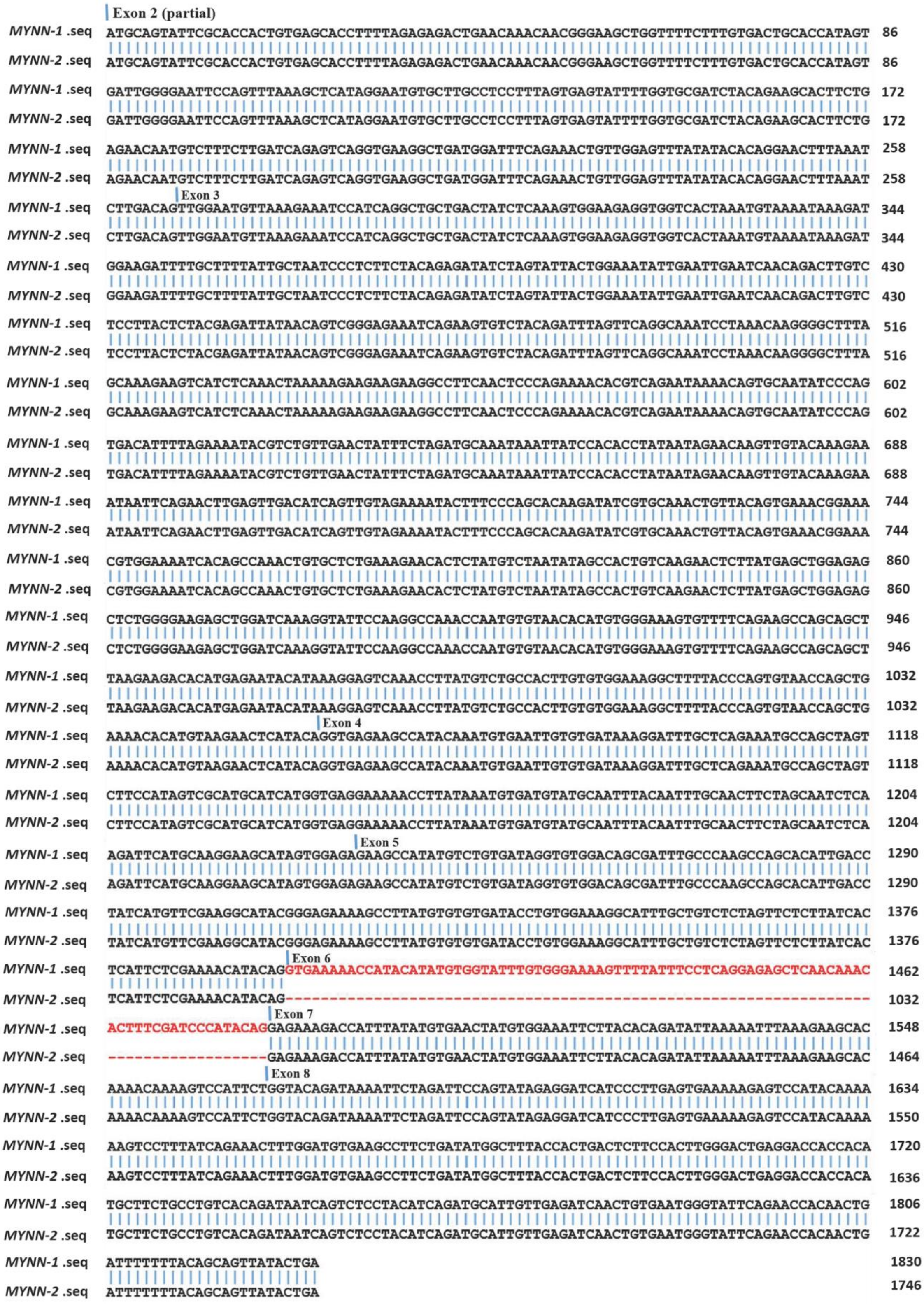

Figure S1. The nucleotide sequence of *MYNN-1* and *MYNN-2*

Note: "*" indicates the same nucleotide; "–" indicates deletion of the nucleotide

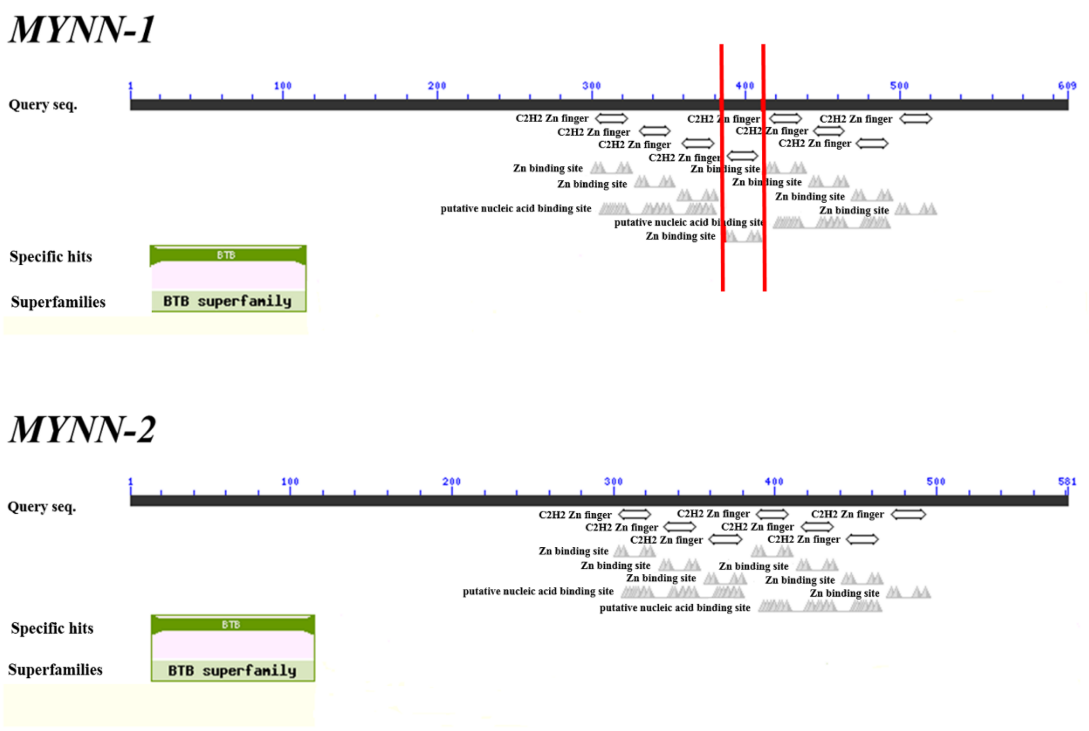

Figure S2. Conserved domain analysis of pig MYNN protein

Note: Double vertical line indicate the difference of predicted domains between MYNN-1 and MYNN-2.
